# In Utero Haematopoietic Stem Cell Transplantation (IUHSCT)

**DOI:** 10.4084/MJHID.2009.031

**Published:** 2009-12-29

**Authors:** Maria Concetta Renda, Aurelio Maggio

**Affiliations:** U.O.C. Ematologia II con Talassemia, Ospedali Riuniti “Villa Sofia-Cervello”, Palermo

## Abstract

In utero haematopoietic stem cell transplantation (IUHSCT) is a non-myeloablative approach for the prenatal treatment of genetic disorders. However, in target disorders, where there is not a selective advantage for donor cells, a useful donor-cell chimerism has not been achieved. There are three possible barriers to engraftment following IUHSCT: limited space in the fetus due to host-cell competition; the large number of donor cells needed, and the immunological asset of recipient.

## Introduction:

Animal models have shown different levels of resistance to IUHSCT engraftment. In primate, goat, rat and mouse the levels of engraftment that has been achieved were low and not therapeutic. Among 46 cases of IUHSCT reported in humans, successful engraftment was obtained only in cases of X-SCID. Useful levels of chimerism has not been achieved in non-immunodeficiency diseases, and a detectable engrafment, was reported only in one case of ß-thalassemia transplanted at 12 weeks of gestation by fetal liver cells. In one α-thalassemia case, where α-globin-dependent hemoglobin production and anemia are present during fetal period, microchimerism and tolerance were suggested.

To overcome the IUHSCT engraftment barriers, it is necessary to develop strategies to improve the competitive capacity of donor cells and to define the gestational age of the possible immunological “window of opportunity” in the human fetus.

In utero haematopoietic stem cell transplantation (IUHSCT) is a non-myeloablative promising approach for the prenatal treatment of a variety of genetic disorders and could be an alternative option to therapeutical abortion in some congenital diseases like haematological hereditary syndromes.

Observations of “naturally” occurring hematopoietic chimerism^1^, and of experimental chimeras produced by IUHSCT, have demonstrated that allogeneic engraftment of the early gestational fetus with donor-specific tolerance is possible^2^.

Although IUHSCT has been clinically successful in severe combined immunodeficiency disease (SCID), a therapeutic donnor-cell chimerism (more than 1%)^3^, useful for the correction of most diseases, has not been achieved in target disorders, like hemoglobinopathies, where there is not a selective advantage for donor cells^4^.

The reasons for this failure are unclear and reflect a limited understanding of the engraftment barriers involved in clinical IUHSCT.

There are three possible barriers to allogenic engraftment following IUHSCT : limited space in the fetus due to host-cell competition from a robust and highly proliferative fetal hematopoietic compartment; the large number of donor cells needed to successfully compete with host cells for these sites; and the immunological asset of recipient that determines the grade of the possible fetal tolerance which is conditional, depending on timing and appropiate presentation of antigen^4^.

The last barrier has been supported by successful allogenic IUHSCT in sheeps and monkeys^5^ occurred if donor and recipient fetuses are in a preimmune condition, by the presence of high-level engraftment after IUHSCT in disorders where exists a competitive or survival advantage for donor cells, and by the presence of VDJ TCR ß chain transcript since the 7^th^ weeks of gestation^6^.

The goal of therapeutically relevant levels of chimerism following a single in utero transplantation has been difficult to achieve. The induction of tolerance with low levels of chimerism to facilitate postnatal cellular transplantations using minimally ablative conditioning regimens is a more realistic goal for clinical application of IUHSCT.

## Cell sources:

Hematopoietic stem cells (HSC) have been isolated from fetal liver^7^, umbilical cord blood, adult bone marrow^8^ and mobilized peripheral blood. HSC isolated from these sources were well described for their capacity to generate mature cells of all lymphohematopoietic lineage and their self-renewal capacity.

All these cell sources were employed to perform IUHSCT; however, if postnatal cellular therapy is needed, the donor cells must be obtained from a renewable source as adult related bone marrow or mobilized peripheral blood^9^.

## Animal models:

An “experiment of nature” first described by Owen in 1945 is the best supporting evidence that IUHSCT might work : dizygotic cattle twins that share cross-placental circulation were born chimeric for their sibilings blood elements, and this state of “chimerism” persisted for life and was associated with specific transplantation tolerance^1^.

The first animal model was the sheep. Early gestational transplantation of allogenic, fetal liver–derived HSC into normal sheep fetuses resulted in a multilineage hematopoietic chimerism^10^ that persisted for many years and ranged from 10% to 15% donor cell expression^11^.

Other normal animal models have shown different levels of resistance to engraftment after in utero transplantation. In the normal primate, goat, rat and mouse (12–16) the levels of engraftment that has been achieved were low and not therapeutic: successful allogenic IUHSCT in monkeys occurred if donor and recipient fetuses are in a preimmune condition^17^.

Almeida-Porada et al. 2000, used the in utero model of human–sheep HSC transplantation to investigate ways of improving engraftment and differentiation of donor cells after transplantation^18^. In this xenogeneic model, the authors co-transplanted HSC and bone marrow-derived human stromal cells obtaining an enhancement of long-term engraftment of human cells in the bone marrow of the chimeric animals and higher levels of donor cells in circulation both during gestation and after birth.

Hayashi et al 2002, obtained the induction of prenatal tolerance in mice where a combined approach of IUHSCTx, performed by intraperitonel injection, followed by postnatal donor lymphocyte infusion has converted low-level mixed hematopoietic chimerism to complete donor chimerism^19^.

A mouse model described by Perenteau et al^20^ strongly support the presence of an immune barrier to allogeneic engraftment after IUHSCT. Four-teen weeks old fetal mice were transplanted with high doses of donor cells of either allogeneic or congenic bone marrow, using intravascular injection technique. Engrafment was lost in 70% of allogeneic recipients by 1 month of age; in contrast, all congenic recipients maintain stable, long-term, multilineage chimerism, thus giving a strong evidence for an innate immune mechanism, natural killer cell or macrophage mediated, able to eliminate allogeneic cells after IUHSCT^20^.

However, further studies are needed to better characterize the timing of human fetal NK-cell maturation.

The reasons for the failure in the correction most diseases and the absence of predicting tolerance in absence of immunosuppression, both in human or animal models, of IUHSCT are still unclear and reflect a limited understanding of the engraftment barriers^21^.

## IUHSCT in Humans:

Since Touraine et al published the first instance of intrauterine transplantation in a human fetus affected by bare lymphocyte syndrome (BLS) in 1989^22^, 46 cases of IUHSCT in human for various indications have been reported^23^ with different timing of transplantation, source of donor cells and the target disease.

However, successful well-documented engraftment after in utero therapy was obtained only in cases of X-linked SCID by Touraine, Flake and Wengler^24^–^26^.

Useful levels of chimerism has not been achieved in non-immunodeficiency diseases, as metachromatic leukodystrophy^27^, chronic granulomatous disease (Flake, unpublished data, 1995) or β-thalassemia major^24^,^27^–^29^.

Particularly, a detectable engrafment, using IUHSCT, was reported only in one case of thalassemia major transplanted at 12 weeks of gestation by fetal liver cells^30^. In this case transitory engrafment was documented by the presence of the Y chromosome of donor origin but not erythroid microchimerism^30^.

Only in one α-thalassemia case, transplanted at 13 weeks of gestation with paternal CD34+ enriched cells, microchimerism and tolerance were suggested^31^. However, the prenatal hematopoietic biology of the α and β-thalassemia are different. In the case of α- thalassemia, α-globin-dependent hemoglobin production (HbF) and anemia are present during fetal period and this could determine a selective advantage for the donor-derived haematopoietic stem cells. Instead, in β-thalassemia the selective advantage could be not present because of globin chain synthesis impairement and anemia do not occur until after birth.

Our experience, in cases treated between the 13^th^ and the 18^th^ weeks of gestation using cryopreserved fetal liver haematopietic stem cells and without immunodepression, was also unsuccessful^29^.

Particularly, a fetus, affected by ß-thalassemia major who underwent a fetus-to-fetus transplantation between the 14^th^ and the 20^th^ week of gestation, generated an alloimmune response as demonstrated by an high cytotoxic T cell precursor (CTLp) frequency against donor cells^32^.

We set-up a clinical protocol by which two female fetuses, 16–20 weeks old, were transplanted with circulating hematopoietic paternal stem cells after one week of low dose dexamethasone treatment with the aim to overcome the engraftment barrier by inducing a transient mild fetal immuno suppression and induce a donor-specific tolerance. During these procedures no adverse events were observed both for the mothers and the fetuses. At birth, both fetuses showed the presence of Y chromosome of donor origin but only one, at two months of age, showed the presence of paternal blood group allele A cDNA in peripheral blood and an increase of HbA concentration from 3% to 14.4%, suggesting that erithroid microchimerism has been occurred. At 5 months of age haemoglobin decreased to 6.8 g/dl and the girl started a regular blood transfusional regimen^33^. This could be explained only by a transitory engraftment of donor cells as it was previously shown in the case described by Touraine^30^.

## IUHSCT: future perspectives:

To overcome the barriers that hinder the success of IUHSCT, it is necessary to develop strategies to improve the competitive capacity of donor cells to achieve significant engraftment, and to define the gestational age of the possible immunological “window of opportunity” in the human fetus.

A significant barrier to allogeneic engraftment following IUHSCT is limited space in the fetus due to host-cell competition from a robust and highly proliferative fetal hematopoeitic compartment in circumstances where a competitive or survival advantage does not exist for donor cells^34^. Additionally, the inability of HSC to home and engraft with absolute efficiency may limit donor-cell engraftment following allogeneic IUHSCT.

Thus, strategies that selectively increase donor HSC homing and engraftment in the fetal recipient may increase both the levels and frequency of engraftment following allogeneic IUHSCT. Dipeptidyl peptidase CD26 inhibition may represent a novel approach to increasing the efficacy and success of HSC/HPC transplantation^35^.

Recent in vitro studies have demonstrated that increased cleavage of stromal-derived factor 1 (SDF-1) by CD26, a chemokine involved in hematopoietic cell chemotaxis, homing, mobilization and survival, results in a loss of its chemotactic effect on primitive hematopoietic cells. Instead, its inhibition results in SDF1–induced migration 36.

In vivo, postnatal studies in a murine bone marrow transplantation (BMT) model found increased short-term bone marrow (BM) homing of enriched HSC and low-density BM donor cells after inhibition or absence of CD26 activity. Reduction of CD26 activity on low-density donor BM cells also resulted in increased long-term engraftment, competitive repopulation, secondary transplantation, and mouse survival following lethal irradiation. Therefore, CD26 inhibition may be an important adjunct to other strategies that are directed toward overcoming the barriers to fetal engraftment^37^. The demonstration that transient manipulation of a single chemokine interaction can result in improvements in engraftment is encouraging, as there are many steps in the homing and engraftment process that could potentially be manipulated, singly or in combination, to significantly improve engraftment^38^.

Other manipulations that selectively influence expression of homing receptors and engraftment include cotransplantation with stroma. The use of stromal cotransplantation has increased short- and long-term donor cell expression in the sheep model^39^,^40^. Human-placenta–derived mesenchymal stem cells (MSC) show a mutilineage differentiation capacity and they have a direct immunosoppressive effect on proliferation of T lymphocytes from peripheral blood and umbilical cord blood. Even if the way that human-placenta – derived MSC modulate the immune system is unclear, this immunoregulatory feature implies that human-placenta–derived MSC have a potential application in allograft transplantation^41^.

Therefore, the recognition of an immune barrier suggests potential immune-based strategies for application to IUHSCT that might prevent loss of engraftment.

Some studies in mice^19^,^42^ support the existence of the phenomenon of fetal tolerance that it may be dependent on timing and appropriate presentation of antigen. Moreover, there are extrathymic^16^ mechanisms of rejection (NK – or B-cell-mediated response) that are poorly understood. In the human fetus there is evidence of immunocompetence from at least the 11^th^ week of gestation suggested by the presence of alloreactive T lymphocytes from the 10^th^ week of gestation that could explain the failure of engraftment in fetuses older than this gestation^6^,^28^,^32^. Therefore, IUHSCT should be performed before the 10^th^ week of gestation. However, at this so early gestation it is impossible to carry out the infusion of CD34 into the umbilical cord by intravascular route or by their infusion into intraperitoneal cavity. One possible future approach could the infusion of these cells into the coelomic cavity.

Coelocentesis at 6–10 weeks of gestation has been successfully used to obtain fetal DNA for very early prenatal diagnosis of genetic diseases^43^–^45^ The celomic cavity is now believed to be an important transfer interface and a reservoir of nutrients for the embryo and it is present since the fourth week after the last menstruation. Therefore, this cavity could be a new route of access to the fetus. In our study of celomic fluid from fetuses sampled from 6.6 to 10 weeks of gestation by coelocentesis we found an immunological pattern showing a very low frequency of the T, pre-B, B lymphocytes, NK and CD34+ cells. Moreover, the analysis of rearranged VDJ TCR β chain transcripts and TCR α chain transcripts showed the presence of only PreTα expression and only 40% (7 of 17) samples showed the presence of some Vβ-subfamilies suggesting that the coelomic cavity could be a useful route of CD34+ cells infusion for “in utero transplantation”^46^.

The injection of donor haematopoietic stem cells into this cavity could potentially induce tolerance in the fetus making successive booster stem cells infusions possible. This procedure has already been evaluated in pre-immune fetal sheep in which the injection of human stem cells through the coelomic cavity was associated with a significant level of engraftment^47^.

## Conclusions:

The intrauterine approach was criticized by several authorities in this field who claimed that this approach did not offer any advantage over postnatal transplantation. The main arguments against intrauterine transplantation were that the fetal invasive procedure carried a certain additional risk.

Conversely, promoters of intrauterine transplantation claimed that reconstitution of the immune system before birth results in reduced susceptibility to infections in the neonatal period, and to an improved psychosocial situation for the family. Other potential advantages for an intrauterine approach include cost savings and a reduced risk of GVHD in the fetal environment.

Thus, a consensus has been difficult to reach and fetal transplantation has not been widely adopted, despite the fact that successful cases treated in utero had outcomes comparable with the best reported with postnatal transplantation^48^.
